# Differences of dynamic responses of single-pile and pile-group foundations in Meizoseismal areas

**DOI:** 10.1371/journal.pone.0354278

**Published:** 2026-07-24

**Authors:** Yunxiu Dong, Zhongju Feng, Jingbin He, Zhen Wang, Bo Wang

**Affiliations:** 1 School of Civil Engineering, Longdong University, Qingyang, Gansu, China; 2 Highway School, Chang’an University, Xi’an, Shaanxi, China; 3 Engineering Experiment Monitoring Institute, Power China Northwest Engineering Co., Ltd., Xi’an, Shaanxi, China; 4 Technology Research Institute, Shandong Road & Bridge Construction Group Co., Ltd., Jinan, Shandong, China; China Construction Fourth Engineering Division Corp. Ltd, CHINA

## Abstract

In order to investigate the dynamic response variations between isolated pile and pile-group systems in high-seismicity regions, we performed extensive shaking table experiments. These tests examined the acceleration characteristics, displacement patterns, and bending moment distributions across single-pile, four-pile, and six-pile configurations when exposed to four different seismic waveforms at a design-level intensity of 0.35g. Additionally, the structural integrity and damage conditions of the pile foundations were systematically assessed. The experimental data revealed that the greatest displacement at the pile head and the highest bending moment values were specifically caused by Kobe wave excitation. Conversely, the El-Centro wave produced the highest acceleration amplifications. The six-pile foundation exhibited optimal resistance to displacement and bending moment, particularly under the 5010 wave, and the single-pile foundation consistently showed the least acceleration amplification. Post-test inspections, including white-noise scanning and visual checks, showed no evidence of significant macroscopic damage across all foundation types, indicating an essentially elastic response at this intensity level. Engineering suggestions for the seismic design of bridge pile foundations in strong earthquake areas are proposed.

## 1. Introduction

Pile foundations are crucial supporting systems for buildings, bridges, and other structures in earthquake engineering. The safety and stability of superstructures are directly influenced by the dynamic behavior of pile foundations during seismic events. Single piles and pile groups, as two main forms of pile foundations, exhibit significantly different dynamic response characteristics. This difference becomes particularly complex under various seismic wave excitations. However, there is a lack of comprehensive understanding of these differences, especially under strong earthquake conditions, which poses challenges for optimizing seismic design and ensuring structural safety.

China, an earthquake-prone country with over 60% of its regions exposed to seismic intensities exceeding 7 degrees, faces significant challenges in infrastructure development [1–3]. The rapid expansion of bridge construction, particularly cross-sea bridges, necessitates foundations in high-seismicity zones. Strong earthquakes frequently cause severe damage to pile foundations, including pile fracture and pier cracking [4–6]. Globally, major seismic events like the 1976 Tangshan (M7.8), 1995 Hanshin (M7.3), and 2008 Wenchuan (M8.0) earthquakes have caused extensive bridge damage [7–9]. More recently, the 2023 Kahramanmaraş earthquake sequence (Mw 7.7 and 7.6) in Türkiye caused liquefaction-induced failure of bridge pile foundations [10], and the 2025 M7.9 Myanmar earthquake led to the collapse of multiple bridges, including the historic Ava Bridge, due to near-fault ground motions and soil liquefaction [11], further highlighting the critical need for research on pile foundation dynamics in strong earthquake areas [12–14].

Against this backdrop, significant research progress has been made using experimental, numerical, and analytical approaches. In centrifuge testing, Liang et al. [15], Garala et al. [7], and Seong et al. [16] used centrifuge tests to link single-pile bending moments to seismic wave spectra and examine monopile foundation responses. In numerical modeling, Bao et al. [17] employed 3D numerical methods to analyze the nonlinear behavior of soil-pile interaction in liquefiable soil, while Radhima et al. [18], Noto et al. [19], and Chen et al. [20] further examined pile-soil-pile mechanisms and modeled interface nonlinearity. Zhao and Zhang [21] investigated the coupling of inertial and kinematic interactions of pile-supported structures in different soil sites through numerical simulations considering soil nonlinearity. In shaking table tests, Chiou et al. [22], Feng et al. [23,24], Zhang et al. [25], and Hu et al. [26] conducted shaking table tests revealing pile spacing effects in soft soil, nonlinear responses, liquefaction behavior, and pile-supported structure dynamics. Jia et al. [27] investigated the seismic bridge-pile-soil system failure mechanisms due to liquefaction-induced lateral spreading through large-scale shaking table experiments, revealing that the liquefaction-induced lateral spreading shifted the vulnerable position of the pile group bridge system from the pier bottom to the pile head. Zheng et al. [28], Feng et al. [29], Zhang et al. [30], He et al. [31], and Liu et al. [32] investigated seismic wave parameter influences and near-fault responses. Wan et al. [33] explored the seismic response of bridge structures in liquefiable sites subjected to near-fault pulse-like ground motions, finding that such motions significantly amplify structural demands compared to far-field records. Regarding pile-soil-structure interaction, Wang et al. [34] and Zheng et al. [35] reviewed its critical importance for seismic design. Al-Khazaali and Vanapalli [36], Rasekh et al. [37] and Zhou et al. [38] expanded knowledge on pile group behavior. Çetindemir [39] provided a comprehensive review of modeling issues on the seismic soil-pile-structure interaction, highlighting the effects of soil nonlinearity and interface conditions.

Furthermore, recent analytical research has advanced the theoretical understanding of pile-soil dynamic interaction. Yang et al. [40] established a coupled vibration model of an unsaturated soil–pile system under Rayleigh waves, revealing that flexible pile-head support conditions significantly influence seismic response. Yang et al. [41] further developed an analytical framework for the dynamic response of piles embedded in unsaturated soil under SH waves, incorporating the effect of superstructure mass. Zheng et al. [42] presented an analytical solution for the vertical dynamic response of pile groups with arbitrary numbers of end-bearing piles in layered soil, explicitly accounting for wave diffraction and scattering effects. Zheng et al. [43] developed a novel model for the kinematic analysis of piles in anisotropic poroelastic soil subjected to vertically incident S-waves, revealing the significant influence of soil anisotropy on pile response. Liu et al. [44] systematically investigated the torsional vibration characteristics of pipe piles in layered unsaturated soil considering the soil plug effect. Zhang et al. [45] studied the seismic response of pile groups embedded in unsaturated soil, considering the coupling of kinematic and inertia pile-pile interactions. Despite these advances, critical gaps persist: most studies focus on single seismic wave types, limiting systematic comparisons between single piles and pile groups. Large-scale shaking table test applications remain scarce, hindering realistic simulation of large bridge foundations. Numerical models struggle with complex nonlinear dynamics, and research on group effect variations under different seismic waves—especially for critical infrastructure like cross-sea bridges—is insufficient.

Therefore, in this study, an artificial mass model was built, and large-scale STTs were carried out to analyze the different dynamic responses between single piles and pile groups under different seismic waves based on pile–soil inertial interactions under strong earthquakes. It is hoped that this research will provide a foundation for the selection of pile types and the seismic design of single piles and pile groups in regions prone to strong earthquakes.

## 2. Project overview

The Haiwen Bridge, the world’s first bridge to cross active faults, is supported on pile foundations. It is located near the epicenter of the 1605 Qiongshan earthquake (M 7.5). The seismic design parameters for the site—a fortification intensity of 8 degrees and peak ground accelerations of 0.35g and 0.59g for 10% and 2% exceedance probabilities in 50 years, respectively—significantly exceed the 0.2g benchmark for Class A bridges in the JTG B02-2013 code. Its seismic design problem is very prominent.

Pier 37 of Haiwen Bridge spans active faults and is located on the hanging wall. The size of the cap is 9.2 m × 9.2 m × 3 m (height), and the left and right caps are connected by tie beams, which are “dumbbell-type” beams. Each cap transfers the load through four 54-meter-long cast-in-place piles (2 m in diameter) that penetrate through over 48 meters of overburden soils—including mucky clay, coarse sand, and gravelly soil—and are socketed into lightly weathered granite for a depth of at least 3*D* (6 m) to ensure stability against seismic and fault displacement effects.

## 3. Shaking table test

### 3.1. Model box

Compared to rigid model containers, a laminated shear box offers superior simulation of soil shear deformation under seismic loading. For such devices, maintaining a length of at least 2 m and a width of no less than 1.5 m ensures that the deviation in vibration frequency remains below 5%, thereby guaranteeing high accuracy. The model box utilized in this experiment measured 3.7 m in length, 2.8 m in width, and 2.0 m in height. It was constructed by assembling 15 layers of hollow steel frames and mounted on a 5.0 m × 5.0 m seismic simulation shaking Table [1].

### 3.2. Theoretical derivation

To ensure the simulation was consistent with the actual project, it was necessary to deduce some parameters theoretically. For example, the geometry and dimensions, constitutive relationship of the material, and mass similarity needed to be consistent with the initial boundary conditions of the model.

In the test, the stress can be expressed by the following equation:


$$\begin{array}{c} \sigma \text{=}f\left(l,E,\rho ,\text{t,r,}v,a,g,\omega \right). \end{array}$$ (1)

This can be transformed into the following dimensionless functional relationship, where the length *l*, density *ρ*, and elasticity *E* are selected as fundamental variables:


$$\begin{array}{c} f\left(\frac{\sigma }{E},\frac{t}{l\sqrt{\rho /E}},\frac{u}{l},\frac{v}{\sqrt{\rho /E}},\frac{a}{E/\rho l},\frac{g}{E/\rho l},\frac{\omega }{\sqrt{E/\rho }/l}\right)=0. \end{array}$$ (2)

Define *λ* as the similarity ratio between the test prototype and the model. According to the dimensional analysis method, the fundamental dimensions of the three independent variables are [l] = L, [ρ] = FL^-4^T^2^, and [E] = FL^-2^. Substituting the dimensions of each parameter in Eq. (2) and setting the π terms of the model equal to those of the prototype yields the similarity relationships for all physical quantities. For example,


$$\begin{array}{c} \lambda \omega =\lambda E\lambda \rho^{-1}\lambda l^{-1}\text{=}1. \end{array}$$ (3)

As both the prototype and the pile foundation model of the STT were constructed with C35 concrete,


$$\begin{array}{c} \lambda \sigma =\lambda E\text{.} \end{array}$$ (4)

With the prerequisite of a constant rigidity of the model pile, based on model mass *m*_*m*,_ the mass *m*_*a*_ is increased to make up for the mass difference *m*_*p*_ between the model and the prototype under the influence of gravity, as follows:


$$\begin{array}{c} m_{a}=\lambda_{E}\lambda_{l}^{\text{2}}m_{p}-m_{m}\text{.} \end{array}$$ (5)

To achieve accurate results, similitude was applied to scale all the necessary material properties of the physical model in this study. Structures were scaled down to smaller sizes. A size similarity ratio of 1/30 was used in this model test, where special iron blocks weighing 25, 100, and 150 kg were rigidly fastened to the center of the pile cap of a single pile, four piles, and six piles, respectively, to serve as artificial masses simulating the inertial effect of the superstructure under seismic loading. Table 1 summarizes the constants of each physical quantity.

**Table 1:** Similarity constants of each physical quantity.

Parameter	Physical quantity	Dimension	Similarity constant	
Load	Acceleration *a*	LT ^− 2^	1	1
	Gravitational acceleration *g*	LT ^− 2^	1	1
	Velocity *v*	LT ^− 1^	C_*l*_^1/2^	0.18
	Time *t*	T	C_*l*_^1/2^	0.18
	Artificial mass *m*_*a*_	FL ^− 1^T^2^	C_*l*_^2^m_*p*_ − m_m_	25 kg/ 100 kg / 150 kg
Geometric characteristics	Length *l*	L	C_*l*_	1/30
	Displacement δ	L	C_*l*_	1/30
	Frequency *ω*	T ^− 1^	C_*l*_^-1/2^	5.48
Material characteristics	Elasticity *E*	FL ^− 2^	1	1
	Stress *σ*	FL ^− 2^	1	1
	Strain *ε*	—	1	1
	Poisson’s ratio *μ*	—	1	1

### 3.3. Experimental design and model preparation

#### 3.3.1. Model design.

In the actual project, the pile for pier 37# is designed to be 54 meters long with a diameter of 2 meters. According to the geometry similarity ratio of the model test (1:30), all model piles were prepared with diameters of 8 cm and lengths of 180 cm, including the single pile a1-1#, four piles b1-1#–b1-4#, and six piles c1-1#–c1-6#. The corner piles are under more unfavorable stress conditions than the middle and side piles; so, they were selected to analyze the differences of the dynamic response characteristics under the different wave types and intensities for the single-pile and pile-group foundations.

**Fig 1:**
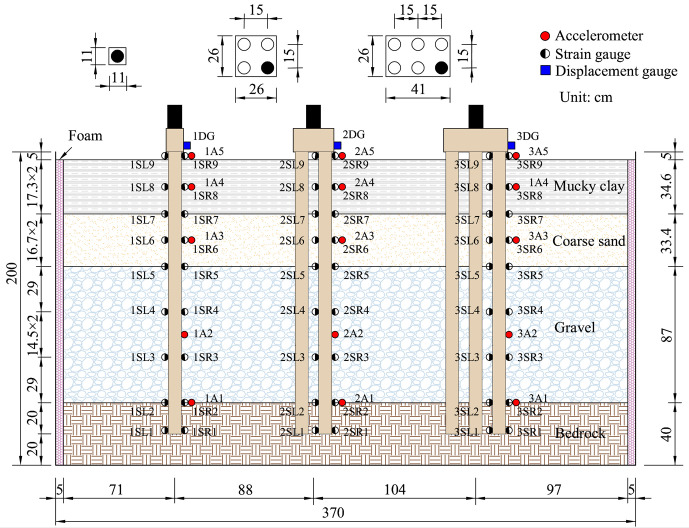
Cross-sectional diagram of the physical model and the placement of instruments showing the soil layer boundaries and thicknesses (unit: cm).

A 50-mm-thick layer of foam was installed along the inner walls of the model box to minimize the impact of seismic wave propagation on its walls, following the practice documented in previous studies [46,47]. Within the model box, piles were arranged in sets of one, four, and six, with each pile spaced 70 cm from the next. The pile spacing of the pile group was 15 cm (about 2D). Sensors, including strain gauges, accelerometers, and displacement transducers, were installed on the piles to monitor their behavior, with the configuration shown in Fig 1.

#### 3.3.2. Model piles.

**Fig 2:**
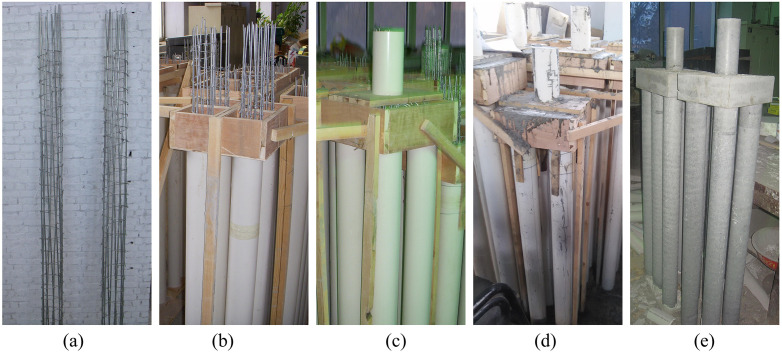
Production process of model pile: (a) Fabrication of reinforcement cage; (b) Polyvinyl chloride (PVC) formwork support; (c) Connection between pile foundation and cap; (d) Erection and pouring; (e) Removal of formwork.

The material of the model piles was concrete C35 with a reinforcement ratio of 2.4%, and steel bars (Q235 type) with diameters of 4 mm were used. The details are presented in Table 2. The production process of the model pile is shown in Fig 2.

**Table 2:** Model pile parameters.

Concrete	Pile length (cm)	Pile diameter (cm)	Bar diameter (mm)	Reinforcement ratio (%)
C35	200	8	4	2.4

**Fig 3:**
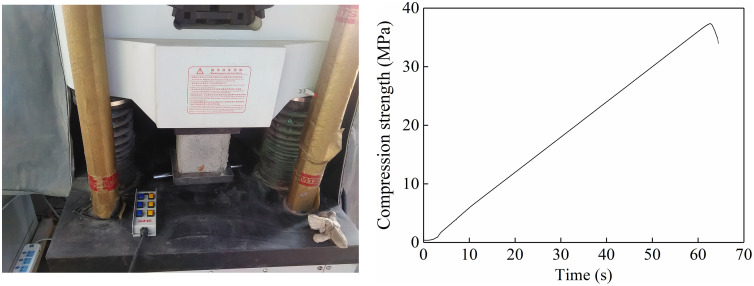
Model pile compressive strength curve: (a) Compression strength test; (b) Compression strength curve.

To check the quality of the model pile, a universal machine was used to determine the compressive strength, and the compressive strength curve is shown in Fig 3. The compressive strength curve showed that the compressive strength of the model pile was 37.4 MPa, which met the test requirements.

#### 3.3.3. Foundation soil.

Based on the controlled shear wave velocity methodology, the model soil was precisely designed to replicate the shear wave propagation characteristics of the prototype soil, thereby ensuring full consistency in dynamic response between the two systems. The prototype foundation supporting the rock-socketed pile was constructed using slightly weathered granite as the bedrock material. Correspondingly, the model bedrock was simulated using C50-grade concrete, which demonstrated an average compressive strength of 50 MPa. During the model bedrock preparation, predefined cavities with a depth of 20 cm were formed at specified positions using PVC pipes having an outer diameter of 8 cm. This configuration allowed for the complete insertion and stable embedding of the model piles into the bedrock [1].

An accelerometer was placed in the lower part of the special closed container, and test soil was filled into the closed container. Then, an accelerometer was placed on the top of the model soil after a fixed level of compaction [1]. The bottom of the closed container was struck, and two accelerometers were used to collect the acceleration response at the same time. The shear wave velocity within the model soil medium is quantified utilizing the fundamental relationship v = Δh/Δt. In this mathematical expression, the variable Δt corresponds to the temporal interval measured between the occurrence of maximum acceleration peaks as the seismic wave propagates vertically through the soil stratum, specifically from the basal interface to the uppermost surface of the soil layer. Simultaneously, the parameter Δh represents the spatial distance traversed by the vibrational wave as it propagates through the geological materials, which includes both rock formations and soil deposits along its transmission pathway.

Once the desired compaction level was reached, direct shear and compression tests were conducted on the soil samples from the model box, and the soil parameters were determined, as shown in Table 3.

**Table 3:** The velocities of shear waves in the soil layers (unit: m·s^−1^).

Type	Mucky clay	Coarse sand	Gravel	Slightly weathered granite/concrete
Prototype	136	263	526	899
Model	138	276	539	917

#### 3.3.4. Seismic waves.

**Fig 4:**
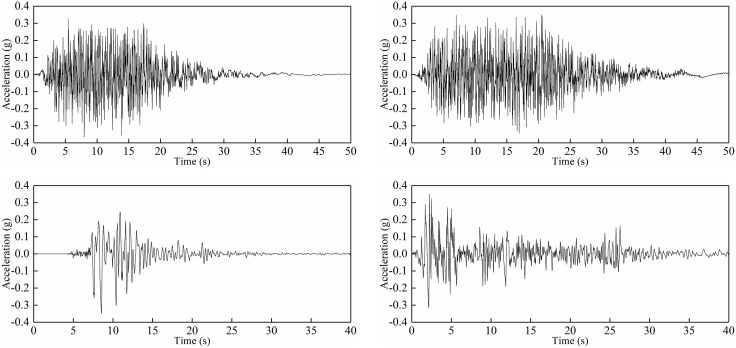
Four different types of seismic waves: (a) 5010 wave; (b) 5002 wave; (c) Kobe wave; (d) El-Centro wave.

Four different types of 0.35g seismic waves were simulated for this test: (a) 10% probability of exceedance in 50 years (5010 wave), (b) 2% probability of exceedance in 50 years (5002 wave), (c) Kobe waves, and (d) El-Centro waves, as shown in Fig 4.

### 3.4. Test conditions

In order to investigate the seismic performance and dynamic behavior of isolated pile foundations, four-pile groups, and six-pile groups when subjected to various earthquake ground motions and different seismic intensity levels, a comprehensive set of experimental conditions was specifically developed for the shaking table tests (STTs), with the complete test matrix being detailed in Table 4. Throughout the experimental procedure, the seismic excitations were implemented incrementally according to increasing intensity levels, and following each seismic wave application, white noise signals were introduced to assess the dynamic properties and potential changes in the structural system’s response characteristics.

**Table 4:** Test conditions.

Pile types	Seismic waveform	Ground motion intensity	Loading directions
Single pile Four piles Six piles	5010 wave	0.35g	X, Y
	5002 wave	0.35g	X, Y
	Kobe wave	0.35g	X, Y
	El-Centro wave	0.35g	X, Y
	White noise	0.05g	X, Y

## 4. Results and discussion

### 4.1. Pile acceleration responses

**Fig 5:**
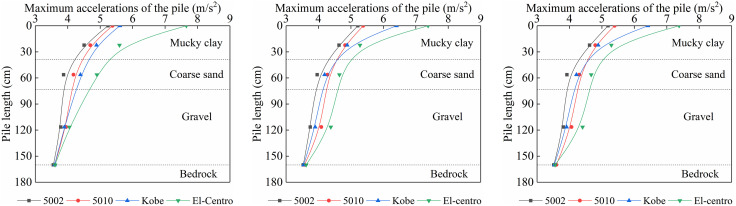
Peak accelerations of different types of pile foundations: (a) Single pile; (b) Four piles; (c) Six piles.

**Fig 6:**
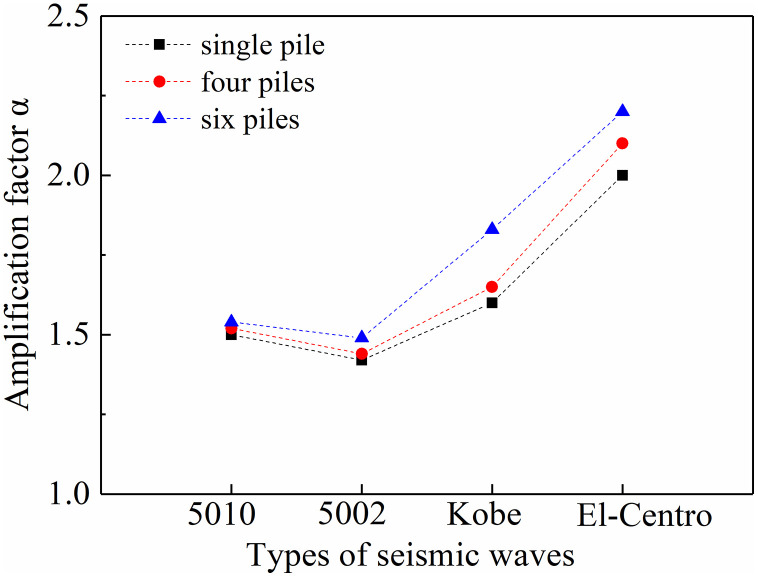
Variations of acceleration amplification factors.


Under the condition where the input seismic ground motion reached a peak acceleration of 0.35g, the dynamic behavior of different pile configurations was systematically analyzed. Fig 5 illustrates the fluctuation patterns in peak acceleration values observed across three distinct pile arrangements: isolated single pile foundation, four-pile group system, and six-pile group configuration (specifically focusing on the corner piles within the group). Concurrently, Fig 6 presents the corresponding variations in acceleration amplification factors throughout the testing process. The acceleration amplification factor, denoted by the symbol *α*, is mathematically defined as the dimensionless ratio obtained by dividing the maximum acceleration response measured at the pile ($a_{\text{pile}}$) by the maximum acceleration of the input ground motion ($a_{\text{input}}$), thereby quantifying the degree of acceleration amplification experienced by the pile foundation system.

$$\begin{array}{c} \alpha =\frac{a_{\text{pile}}}{a_{\text{input}}} \end{array}$$

This represents the amplification effect of seismic waves on the pile top acceleration.

The subsequent information is depicted in Figs 5 and 6:

(1) When subjected to identical levels of seismic wave excitation intensity, the peak acceleration responses exhibited by all three pile foundation configurations demonstrated a consistent pattern of amplification along the vertical profile of the piles. Specifically, the acceleration magnitudes progressively increased from the lower extremities of the pile structures toward their upper sections. Below the interface of the bedrock surface, the acceleration values remained relatively stable without displaying substantial fluctuations or significant variations. However, once above the bedrock boundary, a gradual and continuous upward trend in acceleration amplification was observed, indicating the influence of the overlying soil layers on the dynamic response characteristics of the pile foundation systems. The reason was that the bedrock was made of concrete, which had high rigidity and could transmit earthquake loads well. Therefore, there was no obvious amplification or filtering effect of the seismic waves in the bedrock. At this time, the pile foundation had linear elasticity, but the upper rock–soil exhibited nonlinear properties. The interplay between the inertial effects of seismic wave transmission and the constraint provided by the pile-side soil resulted in a transmitted wave amplitude that was generally larger than the incident wave’s, consequently causing the peak acceleration along the pile to increase gradually.

(2) There were significant differences in the frequencies, cycles, and other parameters for the different seismic waves. Furthermore, the response of the rock–soil system was sensitive to the amplification and filtering effects. As a result, the peak of the acceleration of the pile foundation had the most significant amplification effect in the El-Centro wave (1.85–2.19), whereas the amplification of the amplitude decreased in turn under the Kobe wave, 5010 wave, and 5002 wave. There were also differences in pile top acceleration amplification coefficients of the three types of pile foundations under different seismic waves. The difference in the pile top acceleration amplification coefficients between the six piles and single piles was the largest (0.34) under the El-Centro wave and the smallest (0.03) under the 5010 wave. This showed that the excitation effect was not the same under different types of seismic waves on the same soil layer, and the amplification effect of the acceleration of the pile body under different rock–soil conditions around the pile was also significantly different when subjected to seismic waves. The response was most sensitive to the El-Centro wave.

(3) Under identical seismic excitation, the pile top acceleration amplification factors observed in the six-pile group exceeded those of the four-pile group and the single pile. This phenomenon can be explained by the differing degrees of soil confinement between pile configurations. In the closely spaced six-pile group (spacing = 2D), the soil between adjacent piles is subject to strong geometric confinement, which restricts its freedom to deform under cyclic shear. This confinement limits the build-up of excess pore pressure and reduces the rate of shear modulus degradation compared to the soil around a single pile. As a result, the pile-soil system in the six-pile group maintains a relatively higher overall stiffness and lower effective damping during seismic excitation, providing a more efficient transmission path for seismic waves from the base to the pile top. Consequently, the acceleration amplification factor at the pile top is greater for the six-pile group than for the single pile. Further investigation incorporating direct measurement of soil shear strains between piles would be valuable to provide quantitative validation of this interpretation.

### 4.2. Time-history responses of pile foundation accelerations

**Fig 7:**
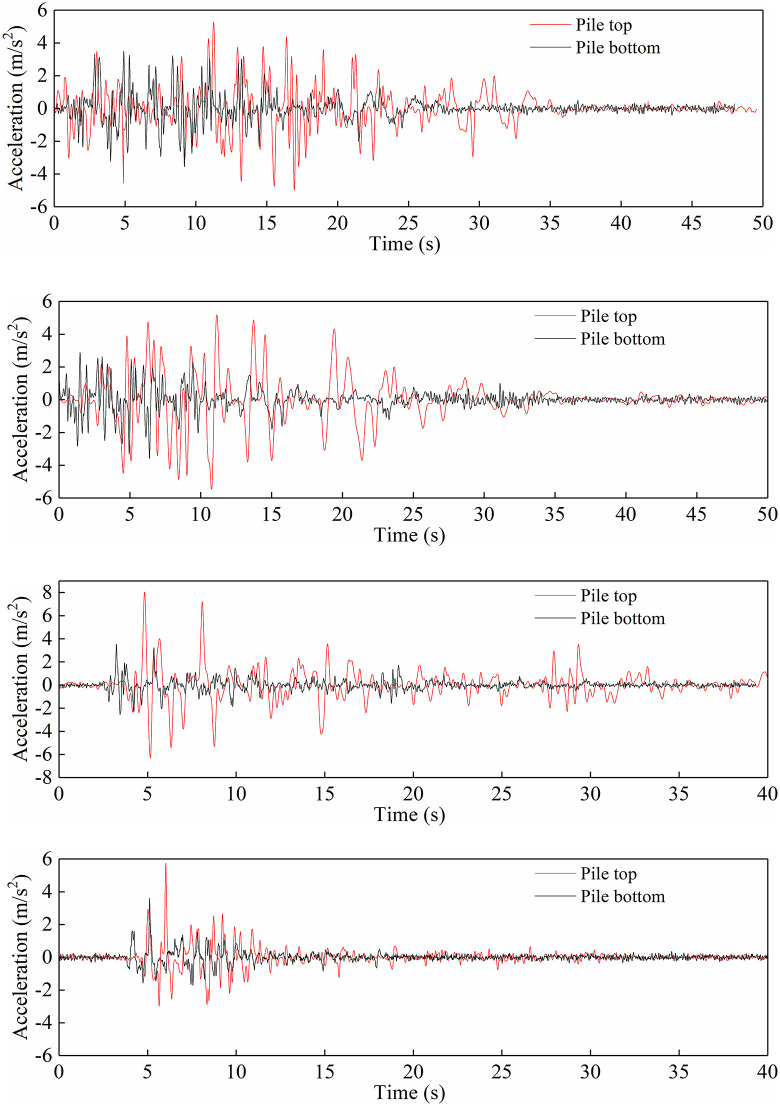
Time-history responses of acceleration on top of six piles: (a) 5002 wave; (b) 5010 wave; (c) EI-Centro wave; (d) Kobe wave.

The six-pile foundation is considered as an example to analyze the acceleration time-history response of the pile foundation, as shown in Fig 7. The times of peak acceleration of pile top and bottom for different types of pile foundations are shown in Table 5.

**Table 5:** Times of peak acceleration of single pile, four piles, and six piles (s).

Pile top/Pile bottom	5010	5002	Kobe	El-Centro
Single pile	11.4/7.1	11.2/9.5	10.4/7.9	8.2/5.3
Four piles	10.9/7.2	11.1/9.4	6.7/5.1	4.8/3.6
Six piles	8.4/4.6	8.7/7.6	6.3/4.6	4.3/3.2
Mean lag time	3.9	1.5	1.9	1.7

The following can be seen from Fig 7 and Table 5:

1. When subjected to various types of seismic wave excitations, the acceleration time-history response at the pile base demonstrated remarkable consistency with the linear variation pattern exhibited by the input seismic acceleration time-history curve. In contrast, the acceleration time-history curve at the pile top displayed a comparatively dispersed distribution pattern, characterized by a reduction in frequency content and an enhancement in amplitude magnitude. This phenomenon can be attributed to the fact that the bedrock material experiences minimal influence from seismic wave frequencies, consequently resulting in the acceleration response at the pile base remaining virtually unaffected. As seismic waves propagate upward from the pile base toward the pile top, the surrounding rock and soil formations act as natural filtering mechanisms for these seismic waves. The nonlinear soil properties effectively filter out high-frequency components during the transmission process of seismic waves, thereby diminishing the acceleration response observed at the pile top.
2. A clear time lag was observed between the occurrence of peak accelerations at the pile top and bottom across all seismic wave types. Here, the lag time is defined as the difference in the absolute occurrence time of the maximum peak in the acceleration time history between the pile top and pile bottom. It should be noted that this definition captures the time difference between the dominant peaks of the entire record, which may correspond to different wave groups rather than the arrival time of a specific seismic phase. Consequently, the measured lag times (1.5–3.9 s) are significantly longer than the time required for a seismic wave to propagate vertically through the model. Based on the model geometry (pile length: 1.80 m with 0.20 m embedment into bedrock; soil layers: 0.346 m mucky clay, 0.334 m coarse sand, 0.87 m gravel) and the measured shear wave velocities in Table 3 (138, 276, 539, and 917 m/s), the theoretical travel time for a shear wave from the pile tip to the ground surface is estimated as t = Σ(hᵢ / vₛ,ᵢ) = 0.20/917 + 0.87/539 + 0.334/276 + 0.346/138 ≈ 0.0055 s. This indicates that the measured lag times reflect not the arrival of the initial seismic wavefront, but the time difference between the maximum peaks of different wave groups that develop in the layered soil model due to multiple reflections at layer interfaces. The single pile exhibited the longest lag time and the six-pile group the shortest across all wave types, a trend consistent with the confinement effect discussed in Section 4.1: the stiffer pile group system responds more rapidly to seismic excitation, resulting in a shorter lag. Comparative analysis revealed that the 5010 wave induced the most significant lag time at the pile top for all pile foundations, with the Kobe, El-Centro, and 5002 waves resulting in progressively shorter lags.
3. In contrast to pile groups, the single pile exhibited delayed peak acceleration times at both its top and bottom, a phenomenon indicative of more pronounced nonlinear interactions with the surrounding rock and soil. Also, the response rate of the seismic wave was slow during transmission, whereas the pile group with rock and soil had good structural integrity and were weakly nonlinear. Furthermore, the velocity was faster during seismic wave transmission.

### 4.3. Displacement responses of pile foundations

**Fig 8:**
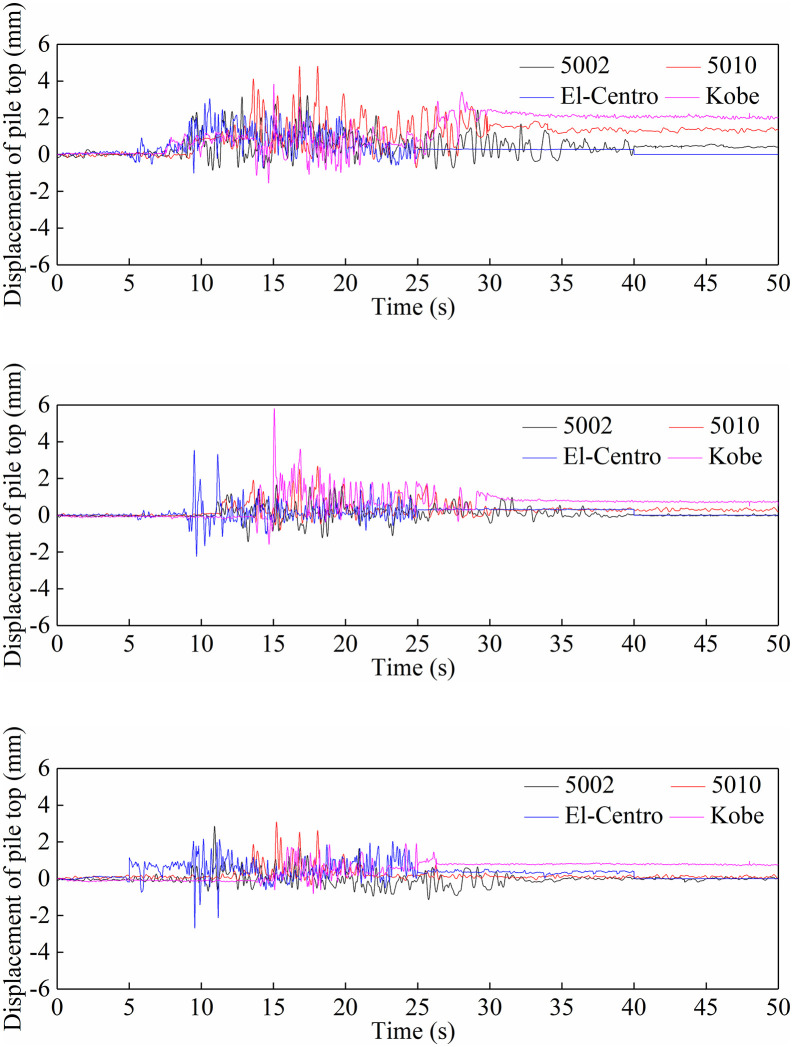
Time-history curve of horizontal displacement of single pile, four piles, and six piles: (a) Single pile; (b) Four piles; (c) Six piles.

The horizontal displacement time-history curves for a single pile, four piles, and six piles under 5010, 5002, Kobe wave, and El-Centro wave loadings are shown in Fig 8.

**Fig 9:**
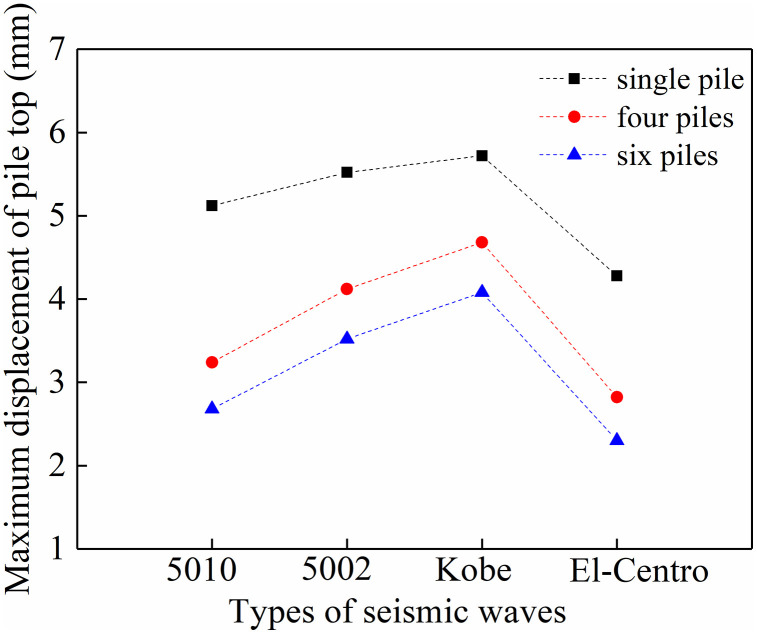
Peak values of horizontal displacements of pile tops.

As the number of piles increased, the amplitude of the pile top’s horizontal displacement decreased, demonstrating the improved lateral resistance of group piles. Taking the Kobe wave as an example, the peak horizontal displacement of the single pile was 5.7 mm, while those of the four-pile and six-pile foundations were 4.7 mm and 4.1 mm, representing reductions of approximately 17.5% and 28.1%, respectively. After vibrations diminished post-40 seconds, displacements stabilized. Notably, permanent displacements were larger under the 5010 and Kobe waves for all foundations, including the single-pile, four-pile, and six-pile types. The peak values of the horizontal displacements of the three types of pile foundations under seismic loading were extracted, and the variations under four types of seismic waves are shown in Fig 9.

Based on the analysis presented in Fig 9, several key observations can be made regarding the performance of pile foundation systems under seismic loading conditions:

Firstly, it is evident that various configurations of pile foundations exhibit consistent patterns in their relative displacement behavior at the pile tops when subjected to different seismic wave excitations. Among the tested seismic waves, the Kobe seismic wave induced the most significant relative displacements at the pile tops, with the maximum horizontal displacement reaching 5.7 mm. In contrast, the El-Centro seismic wave resulted in the minimum relative displacements. This finding suggests that the rock-soil composite system demonstrates heightened sensitivity to Kobe wave excitation, and consequently, the pile top relative displacement attains its peak value under this particular loading condition.

Secondly, through a comparative analysis of the relative displacement characteristics among the three distinct pile foundation types, a clear relationship emerges between the number of piles and the magnitude of horizontal displacement. Specifically, as the quantity of piles in the foundation system increases, there is a corresponding reduction in horizontal displacement. Under the 5010 wave, the six-pile foundation exhibited the largest relative reduction in pile-top displacement compared to the single pile, whereas under the El-Centro wave, the differences among the three configurations were the smallest. However, it is noteworthy that the displacement values recorded for the four-pile and six-pile systems were remarkably similar. This phenomenon can likely be attributed to the minimal variations in pile group effect responses within the respective soil layers for these two configurations, indicating that beyond a certain threshold, increasing the number of piles yields diminishing returns in terms of displacement reduction.

### 4.4. Bending moment responses of pile foundations

**Fig 10:**
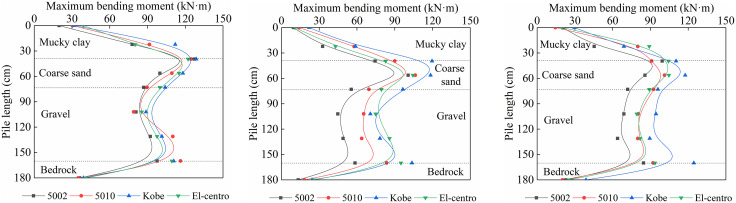
Pile foundation bending moments: (a) Single pile; (b) Four piles; (c) Six piles.

**Fig 11:**
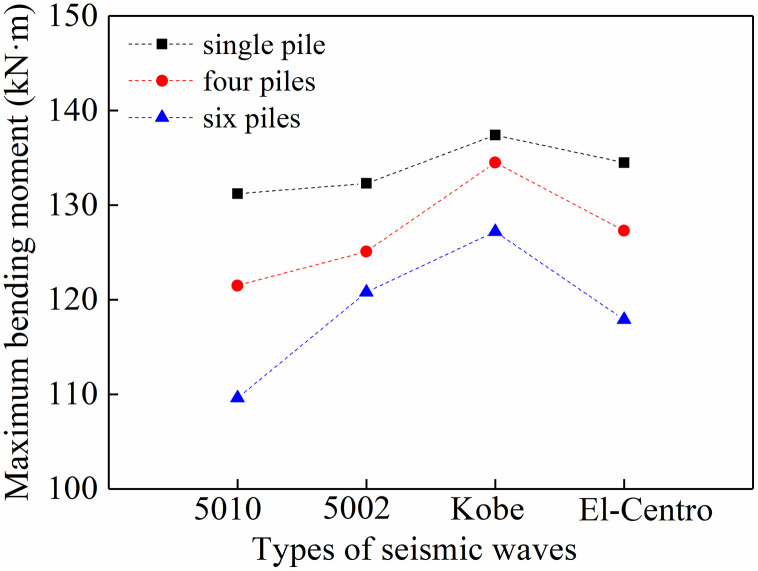
Peak moments of pile bodies.

Fig 10 shows the bending moment responses of the single pile, four-pile, and six-pile foundations under 5010, 5002, Kobe, and El-Centro seismic wave loadings. The pile foundation’s bending moment reaches its maximum variation as shown in Fig 11.

(1) The bending moment responses of the single-pile, four-pile, and six-pile foundations were similar under different types of seismic wave loading. The magnitudes of the bending moment were slightly different and showed double-peak curves along the pile. The maximum bending moments in the pile occurred at depths of 45 cm and 180 cm beneath the pile top. As illustrated in Fig 1 and detailed in the soil profile, the depth of 45 cm aligns with the interface between the coarse sand and gravel layers, while the depth of 180 cm corresponds to the interface between the gravel layer and the concrete bedrock. At these interfaces, the lateral constraint provided by the surrounding soil changes abruptly, resulting in relatively large bending moments. There were differences in the peak bending moment changes between the single-pile, four-pile, and six-pile foundations under the four types of seismic wave loading. The integral moments of the pile foundations were the largest under the Kobe wave loading, followed by those under the El-Centro, 5002, and 5010 wave loadings. This was linked to how the soil’s physical and mechanical properties around the pile responded differently to the seismic wave’s fundamental frequency and wavelength.

(2) When subjected to identical seismic loading conditions, the three distinct pile foundation configurations (single-pile, four-pile, and six-pile systems) exhibited substantial variations in their structural bending moment distributions along the pile body, as well as in their maximum bending moment magnitudes. The recorded maximum bending moment values for these configurations were determined to be 137.4, 134.5, and 127.2 kN·m, respectively. In comparison with the single-pile foundation, the four-pile and six-pile systems demonstrated bending moment reductions of 2.1% and 7.4%, respectively. Furthermore, the damping characteristics associated with pile-soil dynamic interactions showed progressive enhancement during seismic excitation, which was accompanied by the manifestation of pile group effects. From the perspective of structural resistance to bending forces, the six-pile foundation configuration demonstrated superior performance characteristics when compared to both the four-pile and single-pile foundation systems. It should be noted that the pile spacing in this test was fixed at 2D (15 cm), which is close to the lower limit specified in typical bridge design codes and represents a scenario where the pile group effect is highly pronounced. If the pile spacing were increased to 3D or 4D, the soil confinement between piles would be reduced, and the pile group effect would diminish, causing the dynamic response of the pile group to converge towards that of multiple independent single piles. Consequently, the differences in bending moment and other responses between single-pile and pile-group foundations observed in this study would likely decrease with larger pile spacings.

## 5. Seismic damage evaluation of pile foundations

The fundamental frequency of the pile foundation is mainly related to its own quality and stiffness. If the pile foundation is damaged by cracks or other factors, the stiffness of the pile foundation will be reduced, and the fundamental frequency will have significant changes inevitably. Following the completion of each test condition, white noise excitation was applied with incremental increases of 0.05g to systematically investigate the dynamic characteristics of the pile foundations. The acceleration time-history response data collected from the pile body were subsequently processed using the specialized software SEISMO-SIGNAL, which facilitated the generation of Fourier spectrum representations. Through this analytical approach, the natural frequencies of the pile foundation systems were identified and quantified under the influence of various seismic wave conditions, providing valuable insights into the dynamic behavior of the structures.

**Fig 12:**
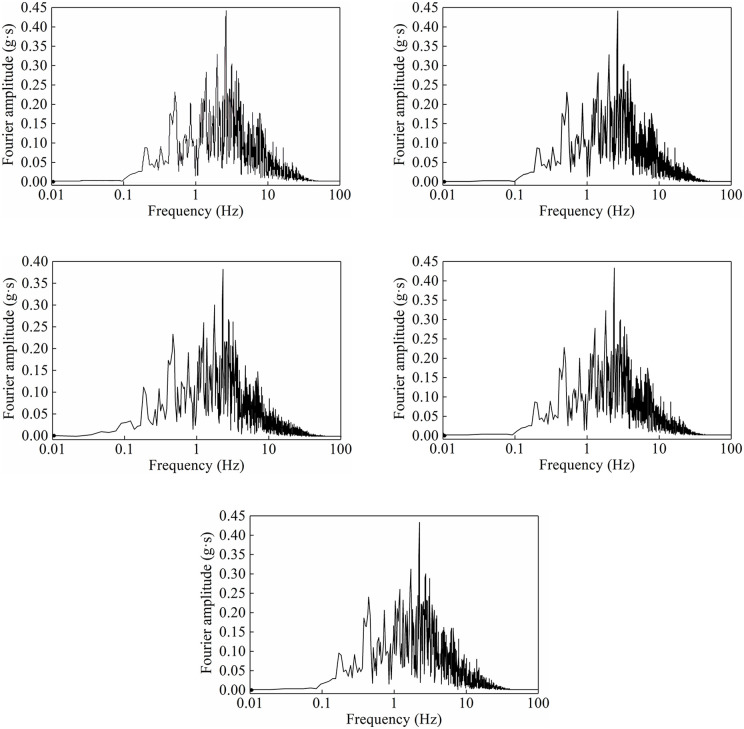
Fourier spectra under different seismic waves: (a) Before the seismic wave input; (b) 5010−0.35g; (c) 5002−0.35g; (d) Kobe-0.35g; (e) El-Centro-0.35g.

**Fig 13:**
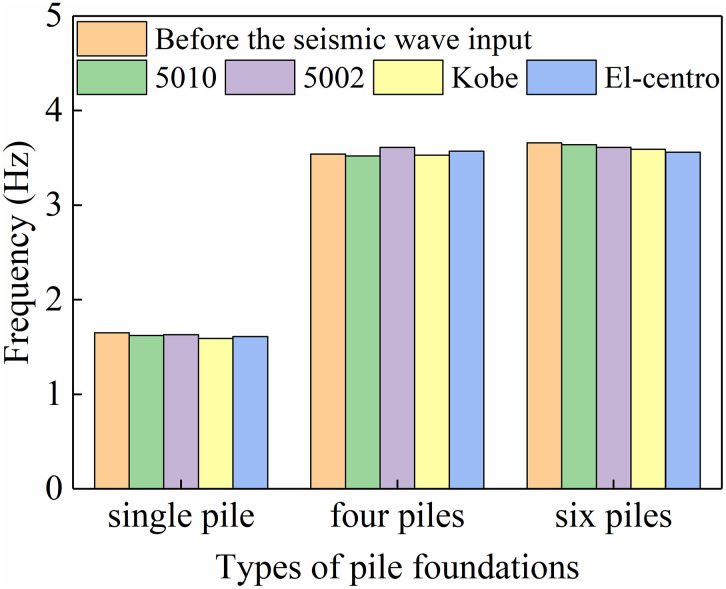
Fundamental frequency changes.

The single pile serves as a case study to assess damage to the pile foundation. Fig 12 displays the Fourier spectra of the single pile subjected to various seismic waves. Fig 13 illustrates the fundamental frequency trends for foundations with one, four, and six piles.

Based on the graphical representations presented in Figures 12 and 13, it can be observed that when subjected to four distinct categories of seismic waves at a peak ground acceleration of 0.35g, the frequency characteristics within the Fourier spectra of the pile foundation systems demonstrated only marginal reduction following the seismic events compared to their pre-earthquake conditions. The absence of substantial alterations in the frequency spectra indicates that the structural integrity and stiffness properties of the pile foundations remained largely uncompromised throughout the seismic loading process. This minimal variation in frequency response suggests that the dynamic characteristics of the foundation systems experienced negligible degradation. Consequently, it can be reasonably concluded that the pile foundation configurations—including the single-pile, four-pile group, and six-pile group systems—maintained their structural stability and did not incur significant macroscopic damage when exposed to the four different seismic wave types at the specified acceleration level of 0.35g. However, it should be noted that damage evaluation based solely on global frequency measurements has inherent limitations; localized minor damage such as micro-cracking may not produce a detectable shift in the fundamental frequency. The absence of visible cracks upon post-test inspection provides additional but not definitive evidence, and the possibility of distributed micro-cracking cannot be entirely ruled out.

## 6. Suggestions

Seismic design proposals should be in line with the above STT results. Based on the STT results, the guidelines for seismic designs are as follows:

(1) The flexural performance of pile foundations is profoundly influenced by the properties of the surrounding soil. Therefore, in the seismic design for strong earthquake regions, particular attention must be paid to the anti-bending design at the interface between soft and hard soil strata, as these are critical zones of potential structural vulnerability.

(2) It is essential to select a seismic waveform that is rationally representative for the design calculations of pile foundations. Analytical results indicate that the El-Centro wave is more suitable for evaluating the peak acceleration response of the pile body, whereas the Kobe wave is more appropriate for assessing the maximum lateral displacement at the pile top and the critical bending moment of the pile body.

(3) The different types of pile foundations have different performances in terms of different response characteristics. The amplification factor of the single pile was the smallest when considering the peak acceleration response of a pile body, and the more piles there were, the larger the amplification factor was. Furthermore, the difference was the largest between the pile groups and the single pile under the El-Centro wave. The lag time of the acceleration response on top of the six piles was the smallest, and the fewer piles there were, the longer the lag time became. The peak value of displacement at the pile top of the six piles was the smallest, as was the pile foundation bending moment. The greatest difference in the piles and pile groups occurred under the 5010 wave excitation. The pile group effect and the most important response characteristic should be considered based on the different types of pile foundations during seismic design and calculations.

## 7. Conclusions

The experimental analysis yielded the following conclusions regarding the seismic behavior of pile foundations:

(1) Waveform Dependency: The input seismic waveform critically governs the response type. The Kobe wave produced the most severe demands for displacement and bending moment, whereas the El-Centro wave generated the highest acceleration amplifications.

(2) Soil Amplification: A significant amplification of seismic waves occurred through the overlying soil, evidenced by higher peak accelerations and a phase delay at the pile top compared to the bottom.

(3) Pile-Group Efficacy: The pile-group effect markedly improved lateral resistance. Increasing the number of piles reduced pile-top displacements and bending moments, with the six-pile foundation performing optimally. Under Kobe wave excitation, the six-pile foundation reduced the peak pile-top displacement by 28.1% and the maximum bending moment by 7.4% compared to the single pile. The largest performance disparity between single piles and pile groups occurred under specific waveforms (El-Centro for acceleration; 5010 for displacement).

(4) Structural Integrity: All foundation models maintained their fundamental frequencies and showed no visible signs of damage, confirming their elastic response within the tested intensity range. However, the possibility of distributed micro-cracking undetectable by the employed methods cannot be entirely excluded.

(5) Limitations and Future Work. Several limitations of this study should be acknowledged. First, the tests were conducted at a single seismic intensity level (0.35g), which was selected based on the design earthquake for the prototype bridge. Multi-level excitation tests are warranted in future studies to characterize the intensity-dependent evolution of the pile group effect. Second, the superstructure was simplified as a concentrated artificial mass rigidly fastened to the pile cap, which primarily captures the first-mode inertial effect and does not fully replicate the soil-pile-superstructure dynamic interaction. Third, the pile spacing was fixed at 2D, representing a scenario of pronounced group effect; the conclusions may not be directly extrapolated to configurations with larger spacings. Fourth, the damage evaluation relied primarily on global frequency measurements and visual inspection, which are not sensitive to distributed micro-cracking. Fifth, an independent quantitative calibration of boundary effects was not conducted. Future research incorporating a range of seismic intensities, pile spacings, a more realistic superstructure model, and refined damage detection techniques would provide a more comprehensive understanding of the differential responses between single-pile and pile-group foundations in meizoseismal areas.

## Supporting information

S1 Supporting information S1 Fig. 1 Cross-sectional diagram of the physical model and the placement of instruments showing the soil layer boundaries and thicknesses. **S2 Fig.** 2 Production process of model pile. **S3 Fig.** 3 Fourier spectra under different seismic waves. **S1 File.** Model pile compressive strength. **S2 File.** Four different types of seismic waves. **S3 File.** Peak accelerations of different types of pile foundations. **S4 File.** Variations of acceleration amplification factors. **S5 File.** Time-history responses of acceleration on top of six piles. **S6 File.** Time-history curve of horizontal displacement of single pile, four piles, and six piles. **S7 File.** Peak values of horizontal displacements of pile tops. **S8 File.** Pile foundation bending moments. **S9 File.** Peak moments of pile bodies. **S1 Table.**
(ZIP)
