# Supplementary material for: Differences of dynamic responses of single-pile and pile-group foundations in Meizoseismal areas
Source: PLoS One. 2026 Jul 24;21(7):e0354278. doi: 10.1371/journal.pone.0354278 (PMC13399318; doi:10.1371/journal.pone.0354278)
Supplement: S1 Supporting information — 1 Cross-sectional diagram of the physical model and the placement of instruments showing the soil layer boundaries and thicknesses. S2 Fig. 2 Production process of model pile. S3 Fig. 3 Fourier spectra under different seismic waves. S1 File. Model pile compressive strength. S2 File. Four different types of seismic waves. S3 File. Peak accelerations of different types of pile foundations. S4 File. Variations of acceleration amplification factors. S5 File. Time-history responses of acceleration on top of six piles. S6 File. Time-history curve of horizontal displacement of single pile, four piles, and six piles. S7 File. Peak values of horizontal displacements of pile tops. S8 File. Pile foundation bending moments. S9 File. Peak moments of pile bodies. S1 Table. (ZIP) [file pone.0354278.s001.zip › supporting information files(1)/S1 Table.pdf]

Table 1. Similarity constants of each physical quantity.

Parameter	Physical quantity	Dimension	Similarity constant	
	Acceleration a	LT^{-2}	1	1
	Gravitational acceleration g	LT^{-2}	1	1
Load	Velocity v	LT^{-1}	$C_l^{1/2}$	0.18
	Time t	T	$C_l^{1/2}$	0.18
	Artificial mass m_a	$FL^{-1}T^2$	$C_l^2 m_p - m_m$	25 kg/ 100 kg / 150 kg
	Length l	L	C_l	1/30
Geometric characteristics	Displacement δ	L	C_l	1/30
	Frequency ω	T^{-1}	$C_l^{-1/2}$	5.48
	Elasticity E	FL^{-2}	1	1
Material characteristics	Stress σ	FL^{-2}	1	1
	Strain ε	—	1	1
	Poisson's ratio μ	—	1	1

Table 2. Model pile parameters.

Concrete	Pile length (cm)	Pile diameter (cm)	Bar diameter (mm)	Reinforcement ratio (%)
C35	200	8	4	2.4

Table 3. The velocities of shear waves in the soil layers (unit: $m \cdot s^{-1}$).

Type	Mucky clay	Coarse sand	Gravel	Slightly weathered granite/concrete
Prototype	136	263	526	899
Model	138	276	539	917

Table 4. Test conditions.

Pile types	Seismic waveform	Ground motion intensity	Loading directions
	5010 wave	0.35g	X, Y
Single pile	5002 wave	0.35g	X, Y
Four piles	Kobe wave	0.35g	X, Y
Six piles	El-Centro wave	0.35g	X, Y
	White noise	0.05g	X, Y

Table 5. Times of peak acceleration of single pile, four piles, and six piles (s).

Pile top/Pile bottom	5010	5002	Kobe	El-Centro
Single pile	11.4/7.1	11.2/9.5	10.4/7.9	8.2/5.3
Four piles	10.9/7.2	11.1/9.4	6.7/5.1	4.8/3.6
Six piles	8.4/4.6	8.7/7.6	6.3/4.6	4.3/3.2
Mean lag time	3.9	1.5	1.9	1.7
