# Supplementary figures and images for: Differences of dynamic responses of single-pile and pile-group foundations in Meizoseismal areas
Source: PLoS One. 2026 Jul 24;21(7):e0354278. doi: 10.1371/journal.pone.0354278 (PMC13399318; doi:10.1371/journal.pone.0354278)

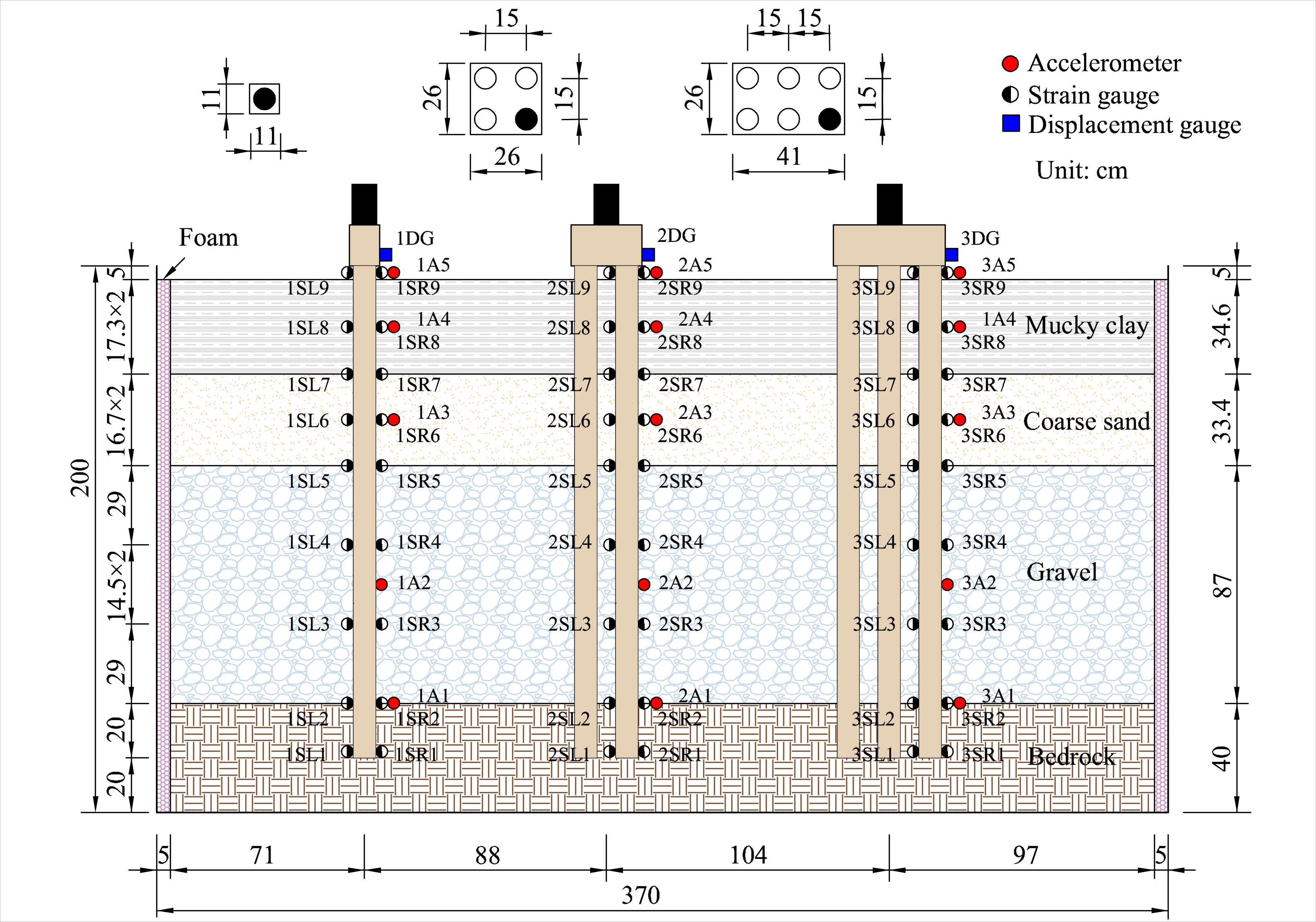

Supplement: S1 Supporting information — 1 Cross-sectional diagram of the physical model and the placement of instruments showing the soil layer boundaries and thicknesses. S2 Fig. 2 Production process of model pile. S3 Fig. 3 Fourier spectra under different seismic waves. S1 File. Model pile compressive strength. S2 File. Four different types of seismic waves. S3 File. Peak accelerations of different types of pile foundations. S4 File. Variations of acceleration amplification factors. S5 File. Time-history responses of acceleration on top of six piles. S6 File. Time-history curve of horizontal displacement of single pile, four piles, and six piles. S7 File. Peak values of horizontal displacements of pile tops. S8 File. Pile foundation bending moments. S9 File. Peak moments of pile bodies. S1 Table. (ZIP) [file pone.0354278.s001.zip › supporting information files(1)/S1 Fig.1 Cross-sectional diagram of the physical model and the placement of instruments showing the soil layer boundaries and thicknesses.tif]

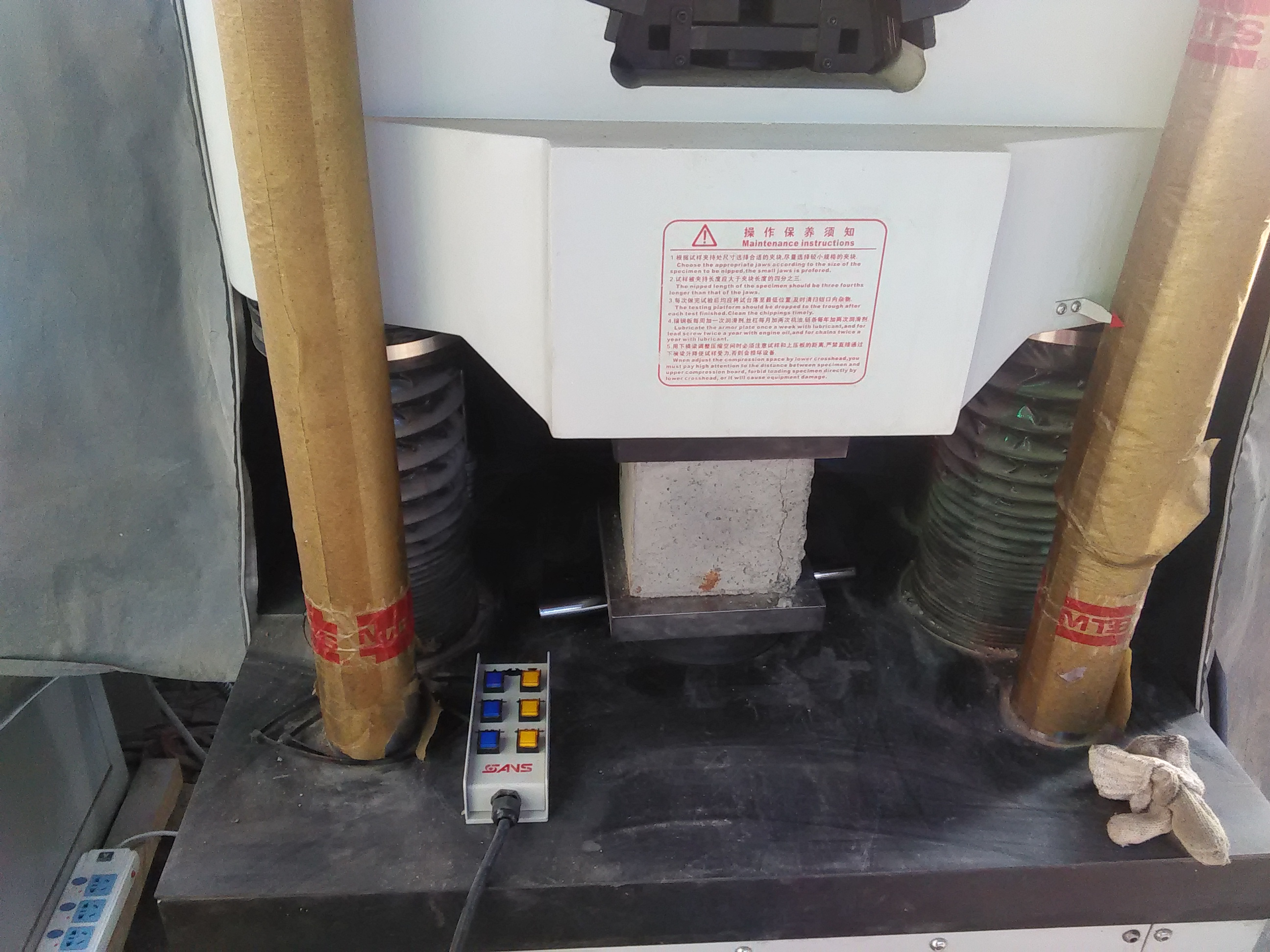

Supplement: S1 Supporting information — 1 Cross-sectional diagram of the physical model and the placement of instruments showing the soil layer boundaries and thicknesses. S2 Fig. 2 Production process of model pile. S3 Fig. 3 Fourier spectra under different seismic waves. S1 File. Model pile compressive strength. S2 File. Four different types of seismic waves. S3 File. Peak accelerations of different types of pile foundations. S4 File. Variations of acceleration amplification factors. S5 File. Time-history responses of acceleration on top of six piles. S6 File. Time-history curve of horizontal displacement of single pile, four piles, and six piles. S7 File. Peak values of horizontal displacements of pile tops. S8 File. Pile foundation bending moments. S9 File. Peak moments of pile bodies. S1 Table. (ZIP) [file pone.0354278.s001.zip › supporting information files(1)/S1 File. Model pile compressive strength/fig3a Compression strength test .tif]

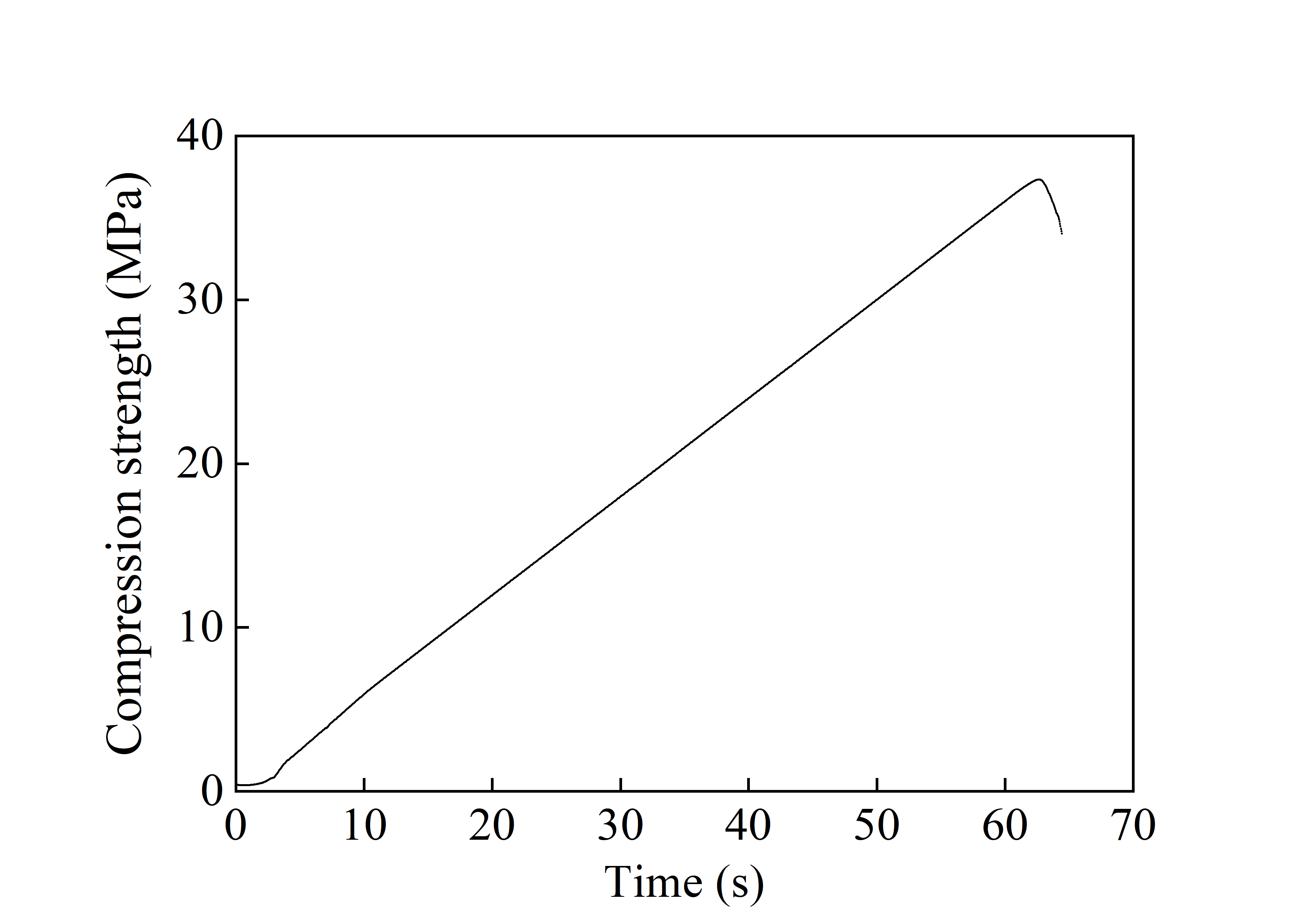

Supplement: S1 Supporting information — 1 Cross-sectional diagram of the physical model and the placement of instruments showing the soil layer boundaries and thicknesses. S2 Fig. 2 Production process of model pile. S3 Fig. 3 Fourier spectra under different seismic waves. S1 File. Model pile compressive strength. S2 File. Four different types of seismic waves. S3 File. Peak accelerations of different types of pile foundations. S4 File. Variations of acceleration amplification factors. S5 File. Time-history responses of acceleration on top of six piles. S6 File. Time-history curve of horizontal displacement of single pile, four piles, and six piles. S7 File. Peak values of horizontal displacements of pile tops. S8 File. Pile foundation bending moments. S9 File. Peak moments of pile bodies. S1 Table. (ZIP) [file pone.0354278.s001.zip › supporting information files(1)/S1 File. Model pile compressive strength/fig3b Compression strength curve.tif]

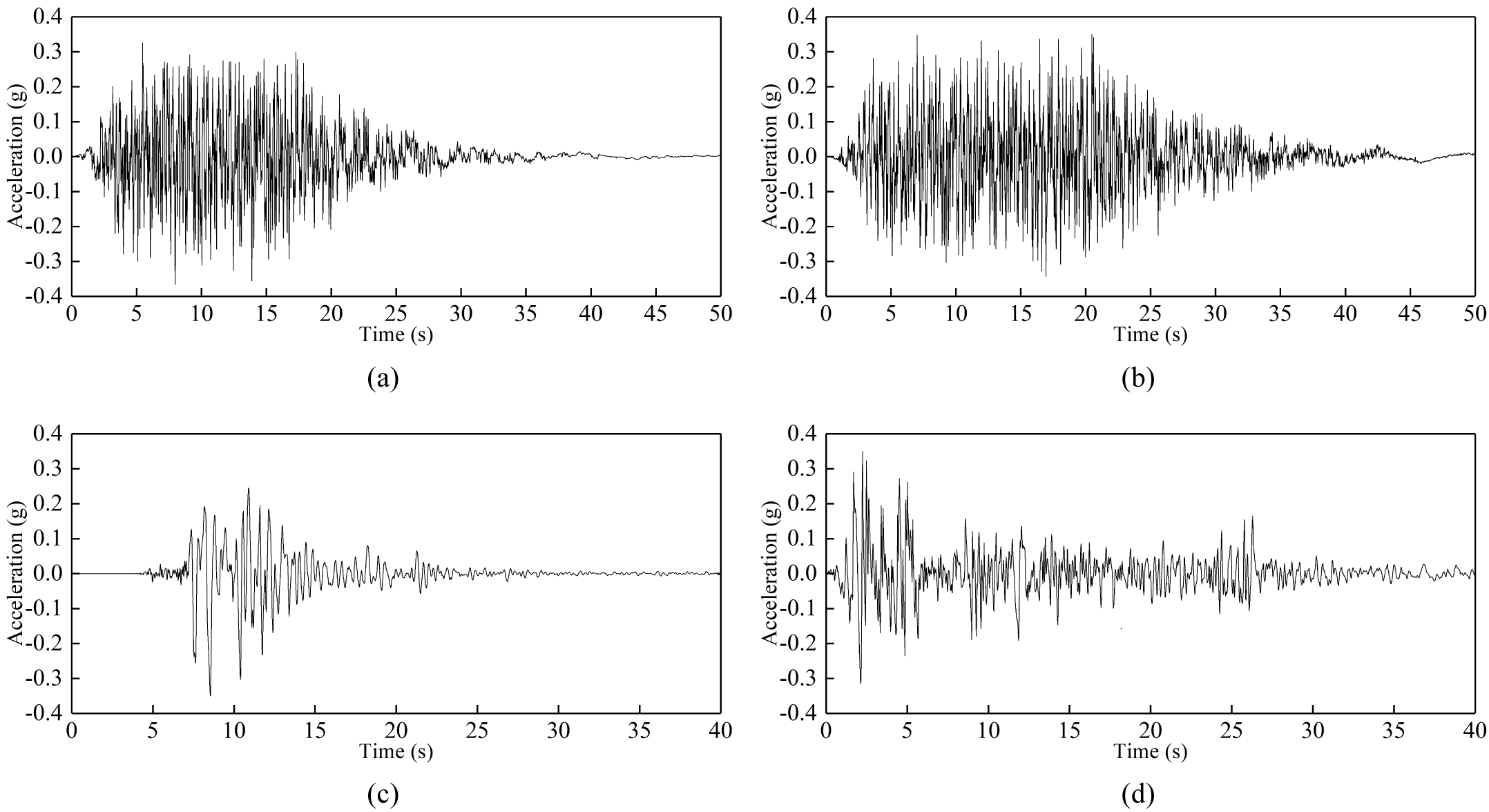

Supplement: S1 Supporting information — 1 Cross-sectional diagram of the physical model and the placement of instruments showing the soil layer boundaries and thicknesses. S2 Fig. 2 Production process of model pile. S3 Fig. 3 Fourier spectra under different seismic waves. S1 File. Model pile compressive strength. S2 File. Four different types of seismic waves. S3 File. Peak accelerations of different types of pile foundations. S4 File. Variations of acceleration amplification factors. S5 File. Time-history responses of acceleration on top of six piles. S6 File. Time-history curve of horizontal displacement of single pile, four piles, and six piles. S7 File. Peak values of horizontal displacements of pile tops. S8 File. Pile foundation bending moments. S9 File. Peak moments of pile bodies. S1 Table. (ZIP) [file pone.0354278.s001.zip › supporting information files(1)/S2 File. Four different types of seismic waves/fig4 Four different types of seismic waves.tif]

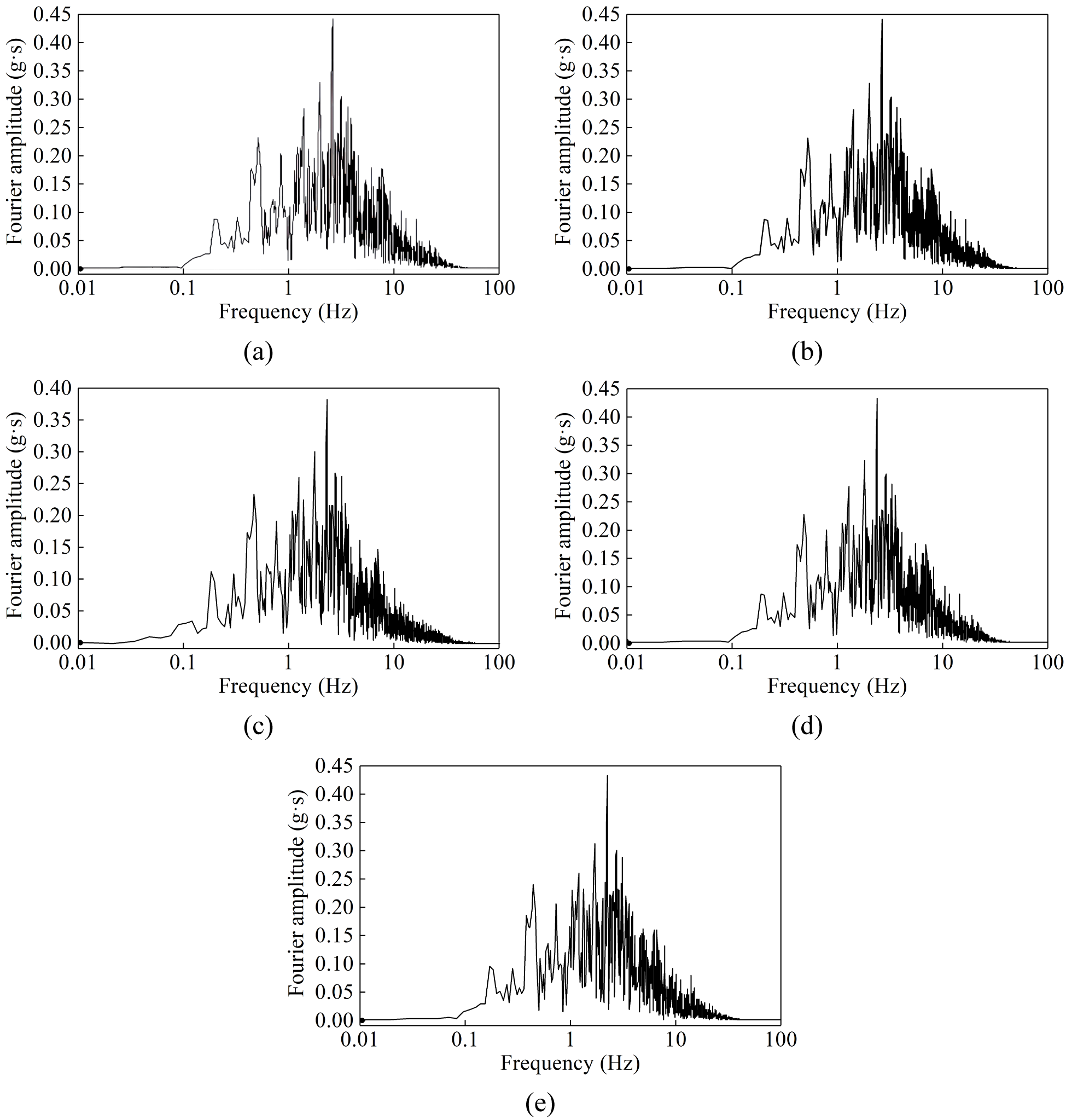

Supplement: S1 Supporting information — 1 Cross-sectional diagram of the physical model and the placement of instruments showing the soil layer boundaries and thicknesses. S2 Fig. 2 Production process of model pile. S3 Fig. 3 Fourier spectra under different seismic waves. S1 File. Model pile compressive strength. S2 File. Four different types of seismic waves. S3 File. Peak accelerations of different types of pile foundations. S4 File. Variations of acceleration amplification factors. S5 File. Time-history responses of acceleration on top of six piles. S6 File. Time-history curve of horizontal displacement of single pile, four piles, and six piles. S7 File. Peak values of horizontal displacements of pile tops. S8 File. Pile foundation bending moments. S9 File. Peak moments of pile bodies. S1 Table. (ZIP) [file pone.0354278.s001.zip › supporting information files(1)/S3 Fig.3 Fourier spectra under different seismic waves.tif]

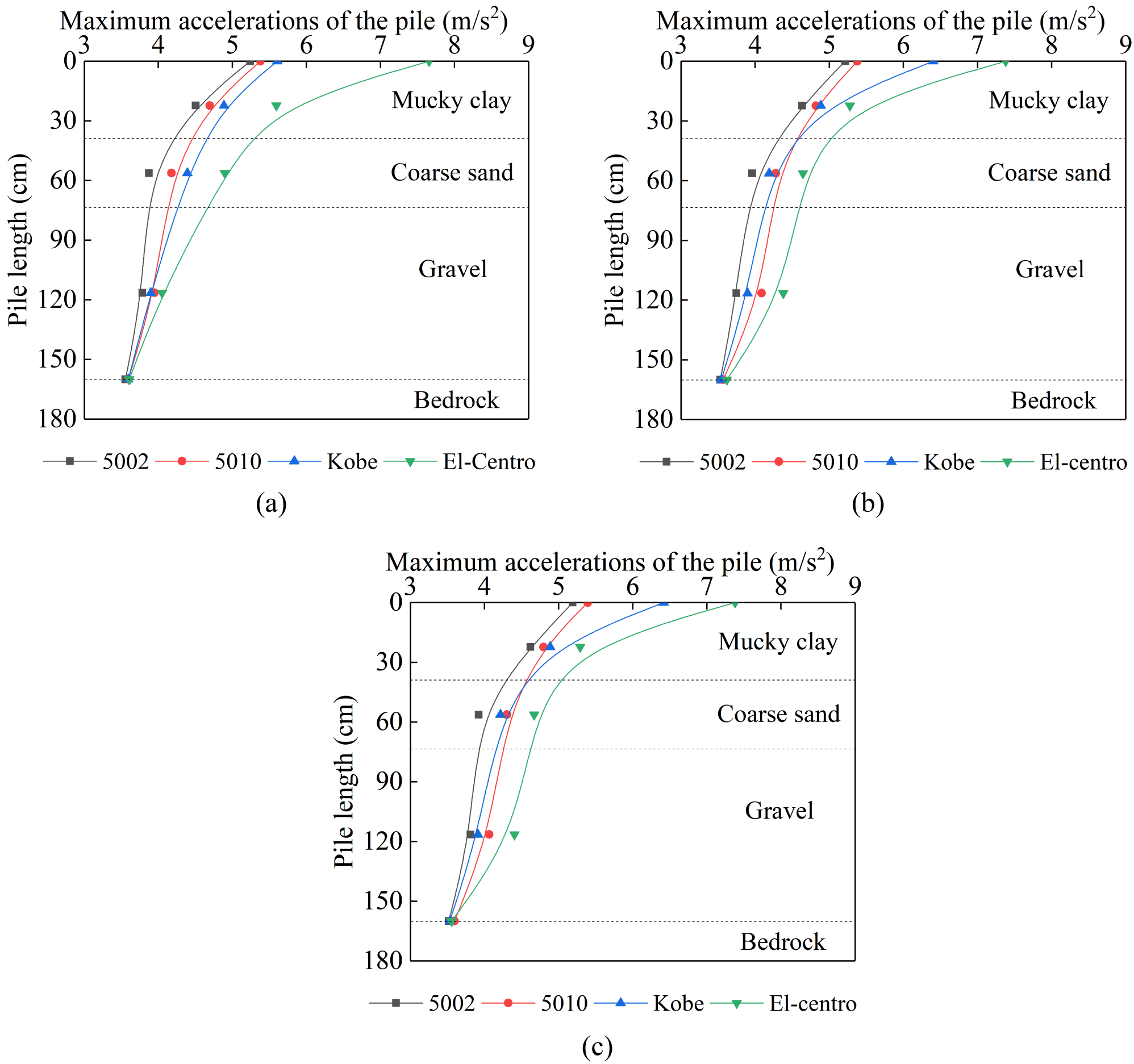

Supplement: S1 Supporting information — 1 Cross-sectional diagram of the physical model and the placement of instruments showing the soil layer boundaries and thicknesses. S2 Fig. 2 Production process of model pile. S3 Fig. 3 Fourier spectra under different seismic waves. S1 File. Model pile compressive strength. S2 File. Four different types of seismic waves. S3 File. Peak accelerations of different types of pile foundations. S4 File. Variations of acceleration amplification factors. S5 File. Time-history responses of acceleration on top of six piles. S6 File. Time-history curve of horizontal displacement of single pile, four piles, and six piles. S7 File. Peak values of horizontal displacements of pile tops. S8 File. Pile foundation bending moments. S9 File. Peak moments of pile bodies. S1 Table. (ZIP) [file pone.0354278.s001.zip › supporting information files(1)/S3 File. Peak accelerations of different types of pile foundations/fig5 Peak accelerations of different types of pile foundations.tif]

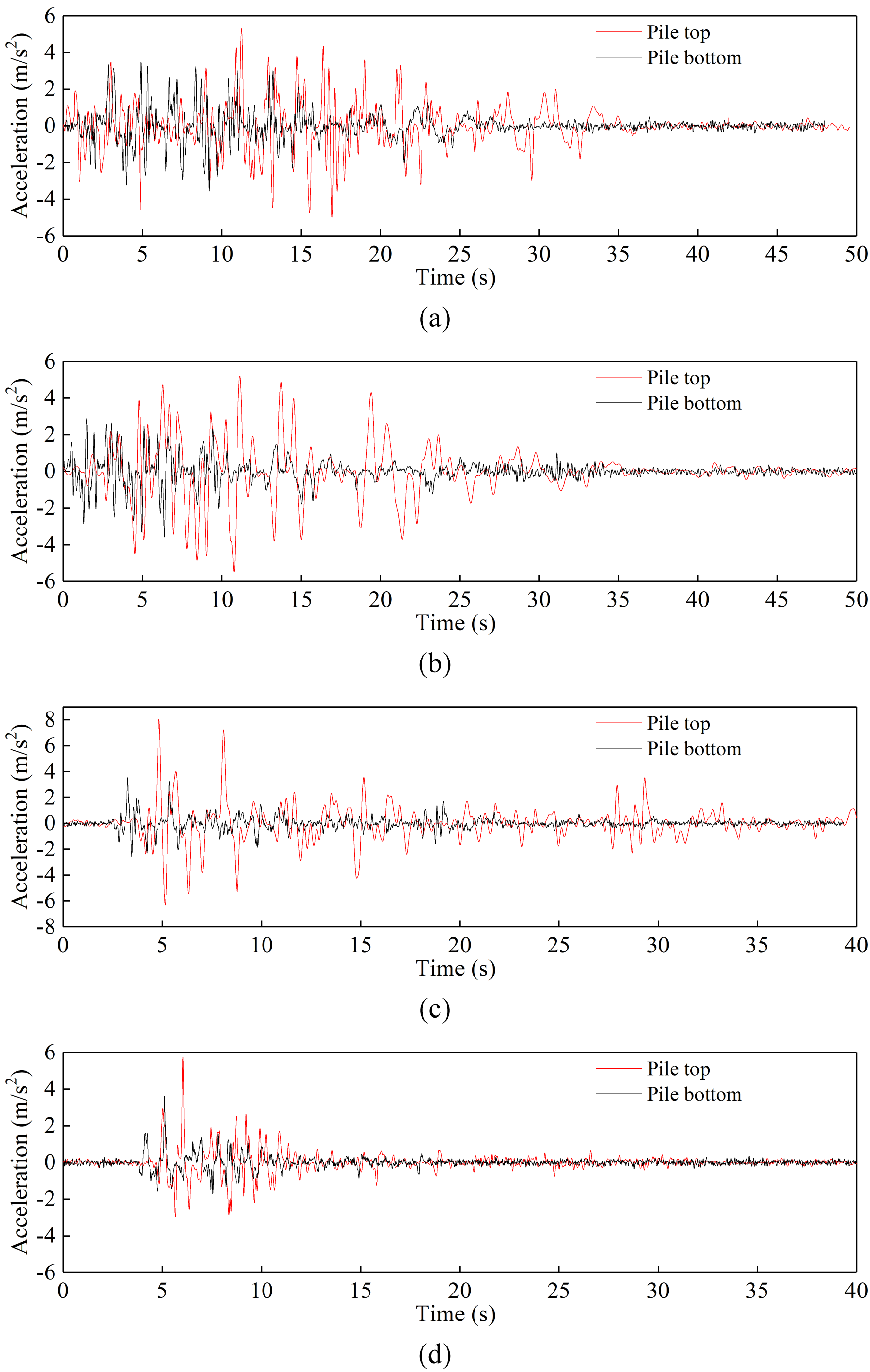

Supplement: S1 Supporting information — 1 Cross-sectional diagram of the physical model and the placement of instruments showing the soil layer boundaries and thicknesses. S2 Fig. 2 Production process of model pile. S3 Fig. 3 Fourier spectra under different seismic waves. S1 File. Model pile compressive strength. S2 File. Four different types of seismic waves. S3 File. Peak accelerations of different types of pile foundations. S4 File. Variations of acceleration amplification factors. S5 File. Time-history responses of acceleration on top of six piles. S6 File. Time-history curve of horizontal displacement of single pile, four piles, and six piles. S7 File. Peak values of horizontal displacements of pile tops. S8 File. Pile foundation bending moments. S9 File. Peak moments of pile bodies. S1 Table. (ZIP) [file pone.0354278.s001.zip › supporting information files(1)/S5 File. Time-history responses of acceleration on top of six piles/fig7 Time-history responses of acceleration on top of six piles.tif]

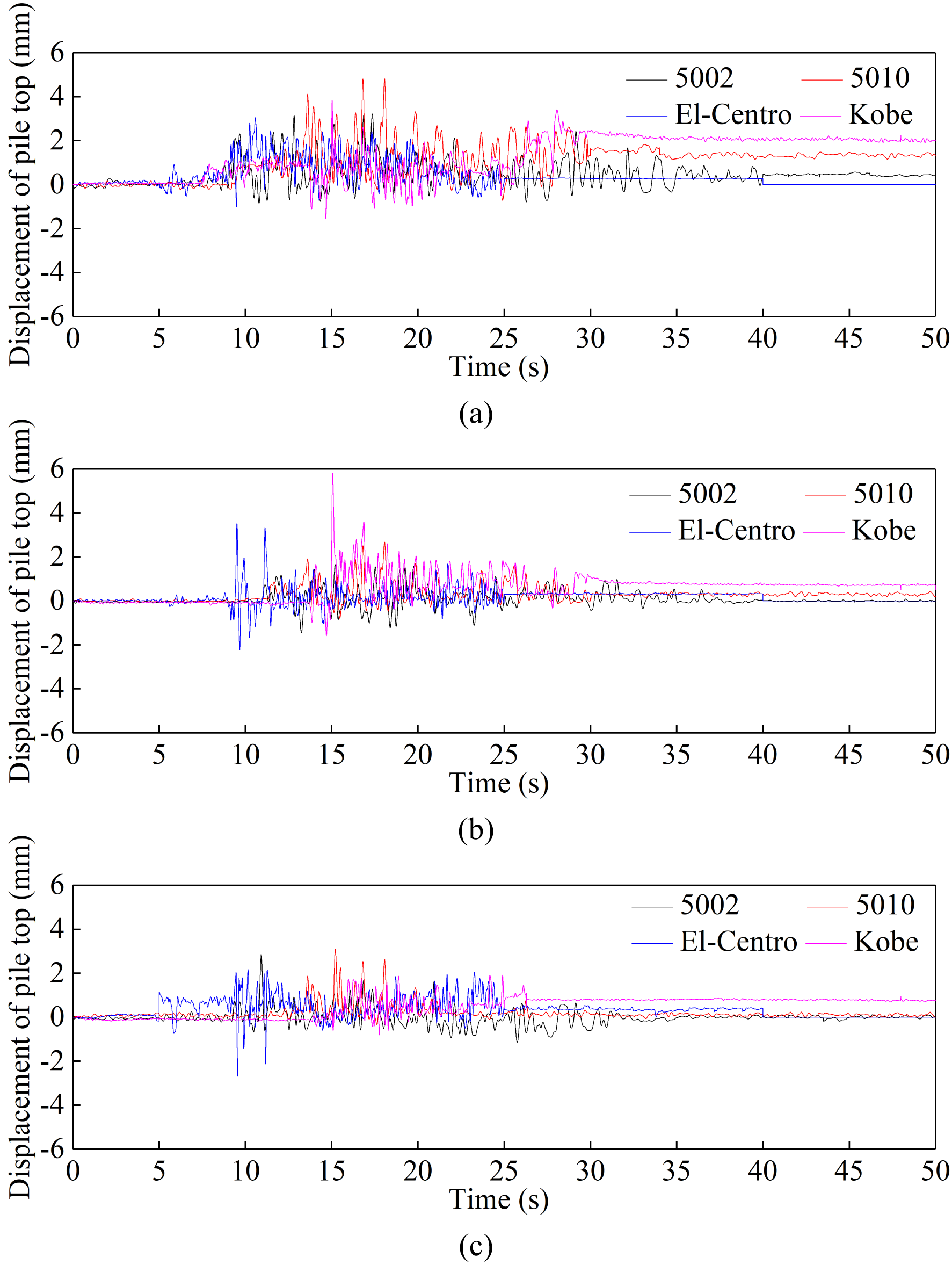

Supplement: S1 Supporting information — 1 Cross-sectional diagram of the physical model and the placement of instruments showing the soil layer boundaries and thicknesses. S2 Fig. 2 Production process of model pile. S3 Fig. 3 Fourier spectra under different seismic waves. S1 File. Model pile compressive strength. S2 File. Four different types of seismic waves. S3 File. Peak accelerations of different types of pile foundations. S4 File. Variations of acceleration amplification factors. S5 File. Time-history responses of acceleration on top of six piles. S6 File. Time-history curve of horizontal displacement of single pile, four piles, and six piles. S7 File. Peak values of horizontal displacements of pile tops. S8 File. Pile foundation bending moments. S9 File. Peak moments of pile bodies. S1 Table. (ZIP) [file pone.0354278.s001.zip › supporting information files(1)/S6 File. Time-history curve of horizontal displacement of single pile, four piles, and six piles/Fig8. Time-history curve of horizontal .tif]

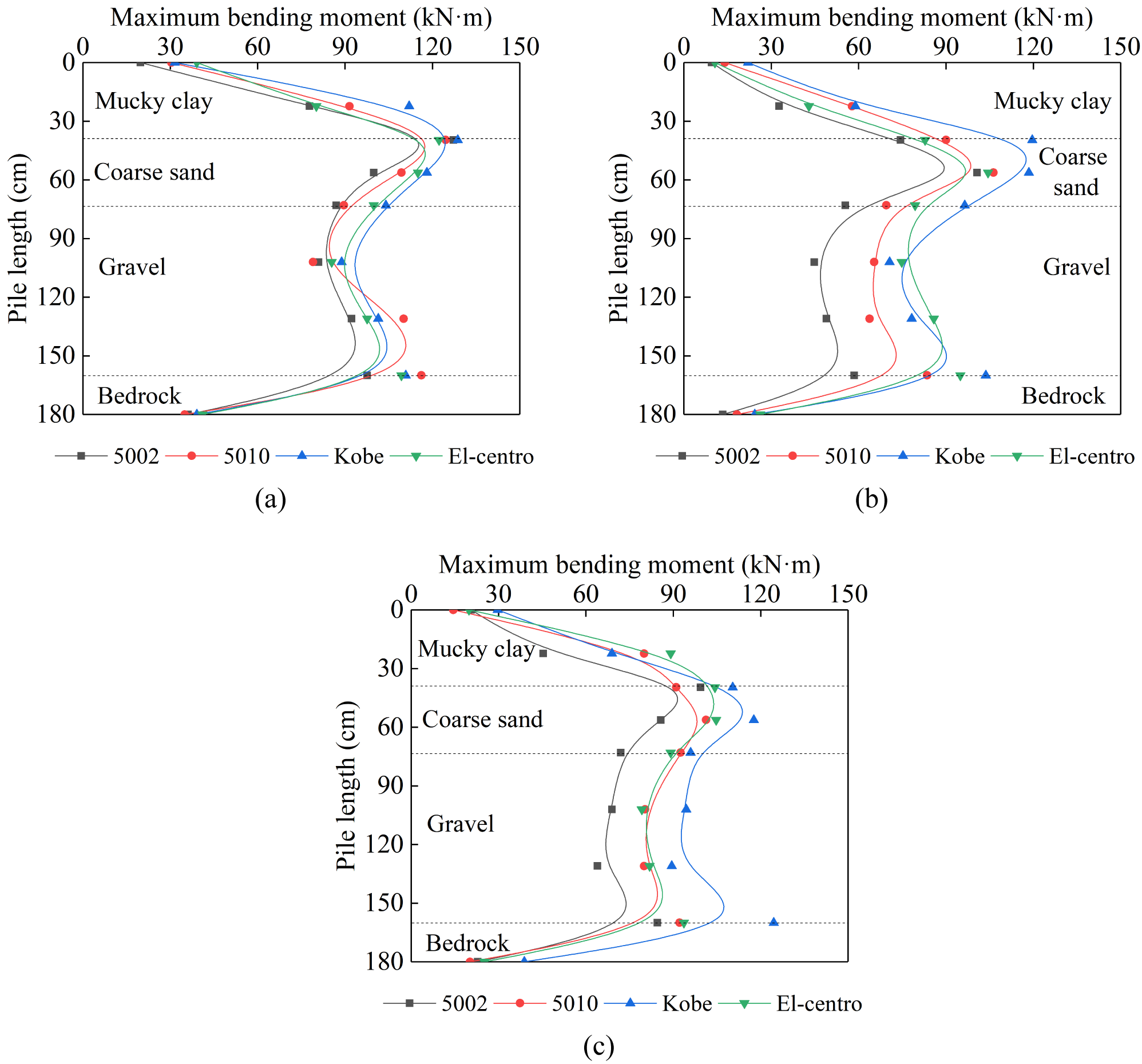

Supplement: S1 Supporting information — 1 Cross-sectional diagram of the physical model and the placement of instruments showing the soil layer boundaries and thicknesses. S2 Fig. 2 Production process of model pile. S3 Fig. 3 Fourier spectra under different seismic waves. S1 File. Model pile compressive strength. S2 File. Four different types of seismic waves. S3 File. Peak accelerations of different types of pile foundations. S4 File. Variations of acceleration amplification factors. S5 File. Time-history responses of acceleration on top of six piles. S6 File. Time-history curve of horizontal displacement of single pile, four piles, and six piles. S7 File. Peak values of horizontal displacements of pile tops. S8 File. Pile foundation bending moments. S9 File. Peak moments of pile bodies. S1 Table. (ZIP) [file pone.0354278.s001.zip › supporting information files(1)/S8 File. Pile foundation bending moments/fig10 Pile foundation bending moments.tif]
